# Development of a High Precision Displacement Measurement System by Fusing a Low Cost RTK-GPS Sensor and a Force Feedback Accelerometer for Infrastructure Monitoring

**DOI:** 10.3390/s17122745

**Published:** 2017-11-28

**Authors:** Gunhee Koo, Kiyoung Kim, Jun Yeon Chung, Jaemook Choi, Nam-Yeol Kwon, Doo-Young Kang, Hoon Sohn

**Affiliations:** 1 Korea Advanced Institute of Science and Technology, Daejeon 34141, Korea; koogh@kaist.ac.kr (G.K.); kiyoungkim@kaist.ac.kr (K.K.); jjysh014@kaist.ac.kr (J.Y.C.); cjmook@kaist.ac.kr (J.C.); 2 Poongsan FNS Corporation, Nonsan 33003, Korea; kwon7020@poongsanfns.co.kr (N.-Y.K.); kdy@poongsanfns.co.kr (D.-Y.K.)

**Keywords:** displacement estimation, Kalman filter, data fusion, RTK-GPS, force feedback accelerometer

## Abstract

A displacement measurement system fusing a low cost real-time kinematic global positioning system (RTK-GPS) receiver and a force feedback accelerometer is proposed for infrastructure monitoring. The proposed system is composed of a sensor module, a base module and a computation module. The sensor module consists of a RTK-GPS rover and a force feedback accelerometer, and is installed on a target structure like conventional RTK-GPS sensors. The base module is placed on a rigid ground away from the target structure similar to conventional RTK-GPS bases, and transmits observation messages to the sensor module. Then, the initial acceleration, velocity and displacement responses measured by the sensor module are transmitted to the computation module located at a central monitoring facility. Finally, high precision and high sampling rate displacement, velocity, and acceleration are estimated by fusing the acceleration from the accelerometer, the velocity from the GPS rover, and the displacement from RTK-GPS. Note that the proposed displacement measurement system can measure 3-axis acceleration, velocity as well as displacement in real time. In terms of displacement, the proposed measurement system can estimate dynamic and pseudo-static displacement with a root-mean-square error of 2 mm and a sampling rate of up to 100 Hz. The performance of the proposed system is validated under sinusoidal, random and steady-state vibrations. Field tests were performed on the Yeongjong Grand Bridge and Yi Sun-sin Bridge in Korea, and the Xihoumen Bridge in China to compare the performance of the proposed system with a commercial RTK-GPS sensor and other data fusion techniques.

## 1. Introduction

Displacement is one of the key physical parameters important to understand the behavior of infrastructure systems. Displacement measurements have been widely used for static and dynamic analysis and design of structures [1,2], structural integrity assessment [3], damage detection [4] and structural control [5]. For example, displacement has been frequently used in bridge loading tests for estimating a bridge’s load carrying capacity and remaining service time [6]. Furthermore, measurement of lateral inter-storey displacement of a building is critical for structural integrity and safety assessment of infrastructure systems during and after earthquakes [7]. For system identification and control, the use of displacement can lead to improved system identification accuracy and structural control efficiency [8].

Many types of sensors and sensing strategies have been continuously introduced into the civil engineering field [9]. However, measuring displacement is still more complex and trickier task than measuring other physical quantities such as acceleration and strain. For example, a linear variable differential transformer (LVDT) measures displacement of a target point by connecting the target point and a stationary reference point [10]. Even though the LVDT is able to measure displacement with high accuracy and at low cost, an additional scaffold is often required for LVDT installation and its application is limited to small-scale structures. Laser-based sensors including laser Doppler vibrometers (LDV) utilize a laser beam aimed at a target point for high fidelity displacement measurements [11]. However, LDV is not commonly used for infrastructure monitoring since LDV can only measure out-of-plane displacement, and the incident angle of the laser beam has to be close to normal to the target surface, and this condition is often difficult to achieve. Also, the LDV is rather expensive (over $100,000 USD), therefore, it is not advised to leave LDV unattended for an extended time period. Vision-based sensors are low cost and can measure displacement from multiple target points simultaneously [12,13,14]. However, the vision-based sensors are mainly used for in-plane measurements and highly affected by ambient light and weather conditions. In addition, the current displacement sensors share a few common limitations, which make them less attractive for long-term monitoring of large-scale civil infrastructure: (1) Securing uninterrupted line of sight becomes challenging for long term monitoring due to daily and seasonal variation of light conditions, weather, raining, fog, etc. Furthermore, it is difficult to secure a line of sight for locations like the top of a bridge pylon and the roof of a high-rise building. (2) Because a rigid ground support or a reference point with negligible displacement is necessary, these sensors are not applicable during earthquakes.

In recent years, real-time kinematic global positioning system (RTK-GPS) devices have been widely used for civil infrastructure monitoring [15,16,17] addressing the first two common limitations mentioned in the previous paragraph. However, RTK-GPS still has room for improvement: (1) the accuracy of RTK-GPS is about 1–4 cm horizontally and 2–8 cm vertically [18], (2) the sampling rate of RTK-GPS is restricted to at most 20 Hz [19], (3) the measurement quality of RTK-GPS can be easily deteriorated due to multipath [20] and blocking of satellite signals, and weather conditions [21], and (4) the price of a RTK-GPS sensor is about $30,000 USD, and it is thus considered rather expensive.

Another approach to measure displacement is to fuse data from multiple sensors. Chan et al. [22] utilized an empirical mode decomposition adaptive filter to estimate displacement by fusing acceleration and displacement measured by an accelerometer and a RTK-GPS. Because displacement is estimated by post-processing the sensor data after all measurements are complete, this technique is not suitable for real time monitoring. Park et al. [23] estimated displacement by combining acceleration and strain measurements and solving an optimization problem formulated by Hong et al. [24]. The technique requires a finite element model of a structure to convert strain measurement to displacement. In addition, parameters for the optimization should be determined manually, and displacement can be estimated only after all measurements are finished, making this technique less attractive for continuous monitoring. Note that real-time estimation is essential to continuously assess the integrity and safety of the monitored structure and to alarm abnormal behavior in a timely manner.

For autonomous and real-time displacement estimation, a Kalman filter-based displacement estimation technique was first proposed by Smyth and Wu [25]. The technique fuses acceleration and displacement measurements, and applies Rauch-Tung-Striebel (RTS) smoothing to enhance its estimation quality. However, this technique does not explicitly consider a bias term in the acceleration measurement. Therefore, the bias tends to accumulate over time when acceleration measurement is double integrated to estimate displacement. To address this problem, the bias term in acceleration measurement is considered in a state-space model of Kalman filtering [26,27]. These Kalman filter-based techniques improve displacement estimation accuracy, but still require Kalman filter smoothing, which hinders real-time displacement estimation due to their large computational demands. Kim et al. [28] proposed a new Kalman filter technique without any smoothers so that displacement can be efficiently and accurately estimated with little time delay.

In this study, a new displacement measurement system is proposed for continuous real-time measurement of displacement, velocity and acceleration of large-scale civil infrastructures. The proposed system can be applied to static, pseudo-static or dynamic displacement measurements of various civil infrastructure systems. Recently, many large-scale structures such as cable-stayed and suspension bridges are instrumented with both RTK-GPS and accelerometers. Using the proposed system, a single sensor module can be used for simultaneous measurements of displacement and acceleration as well as velocity. The proposed system is composed of three modules—a sensor module, a base module, and a computation module. By fusing a low cost RTK-GPS receiver and a force feedback accelerometer, the proposed system provides high accuracy displacement measurement with a high sampling rate. The novelty of the proposed measurement system lies on the followings: (1) 3-axis displacement as well as acceleration and velocity can be measured in a single sensor unit in real-time, (2) displacement is sampled at 100 Hz, which is five times of the sampling rate of a commercial RTK-GPS system, (3) the accuracy of displacement measurement is around 2 mm in a vertical direction, which is at least 10 times better than 2–8 cm accuracy of a conventional RTK-GPS, and (4) the proposed system is highly cost-effective compared to commercial RTK-GPS.

This paper is organized as follows: Section 2 briefly reviews the principle of RTK-GPS, and Section 3 describes the sensor, base and computation modules that make up the proposed system. The performance of the proposed system is validated through lab scale tests in Section 4. Field tests are performed at three different bridges sites, and the performances of the proposed system and a commercial RTK-GPS are compared in Section 5. Finally, the conclusion and discussions are provided in Section 6.

## 2. Introduction to RTK-GPS

In this section, the standard GPS and RTK-GPS are briefly reviewed for the completeness of the paper. A GPS receiver accepts carrier signals from multiple satellites. Various information such as clock time, position and velocity parameters of satellite are transferred to the receiver through the carrier signals [29]. The receiver estimates the distance from a satellite to the receiver by computing the phase shift between the reference signal in the receiver and the carrier signal from the satellite, and the absolute latitude, longitude and altitude of the GPS receiver are provided. Its horizontal and vertical accuracy is 3–5 m and 12–15 m, respectively. The measurements by a GPS have several sources of errors such as clock error of satellites and GPS receivers, ionospheric and tropospheric delays, and multipath error [30,31]. A carrier phase *ϕ* of a standard GPS can be expressed as:

(1) $$\phi =\rho +I-Tr-c\left(b-b_{sat}\right)-\lambda N+\epsilon_{\phi }$$

where ρ is the geometric distance between the satellite and an antenna of the GPS receiver, λ the carrier wave length of a satellite, I and Tr ionospheric and tropospheric delays, b and $b_{sat}$ GPS receiver and satellite clock errors [32], N an ambiguity integer, c the speed of light, and $\epsilon_{\phi }$ a multipath error, respectively. Multipath means that a carrier signal from a satellite can reach the GPS receiver through multiple paths due to reflections from surrounding obstacles, and this multipath can produce 1–2 cm error. Note that Equation (1) does not consider the discrepancy between the antenna phase center (APC) and the antenna geometric center [33], referred to as antenna center tightness. This antenna center tightness depends on the incident angle of the carrier signal with respect to the antenna, and many commercial GPS antennas limits the antenna center tightness within 1 mm.

While a standard GPS relies only on a carrier signal received by a single GPS receiver, RTK-GPS uses two GPS receivers (i.e., base and rover) and obtains a relative 3D position (north—east—depth) of the rover with respect to the base with 1–4 cm horizontal and 2–8 cm vertical accuracy. RTK-GPS can improve displacement estimation accuracy by cancelling some of the error terms in Equation (1).

Let a base *α* be placed to a fixed ground, and a rover *β* be attached on a target measurement point. Then, Equation (1) for the base and the rover can be written as:

(2) $$\phi_{\alpha }=\rho_{\alpha }+I_{\alpha }-Tr_{\alpha }-c\left(b_{\alpha }-b_{sat}\right)-\lambda N_{\alpha }+\epsilon_{\phi ,\alpha }$$

(3) $$\phi_{\beta }=\rho_{\beta }+I_{\beta }-Tr_{\beta }-c\left(b_{\beta }-b_{sat}\right)-\lambda N_{\beta }+\epsilon_{\phi ,\beta }$$

When the base and the rover are close to each other, $I_{\alpha }$ and $I_{\beta }$, and $Tr_{\alpha }$ and $Tr_{\beta }$ can be assumed to be identical. Then, these error terms can be cancelled out by subtracting Equations (2) and (3):

(4) $$\Delta \phi_{\beta \alpha }=\Delta \rho_{\beta \alpha }-c\Delta b_{\beta \alpha }-\lambda \Delta N_{\beta \alpha }+\Delta \epsilon_{\phi ,\beta \alpha }$$

In addition, when the base and the rover receive satellite signals from two satellites denoted as i and j, Equation (4) for satellites i and j become:

(5) $$\Delta \phi_{\beta \alpha }^{j}=\Delta \rho_{\beta \alpha }^{j}-c\Delta b_{\beta \alpha }-\lambda \Delta N_{\beta \alpha }^{j}+\Delta \epsilon_{\phi ,\beta \alpha }^{j}$$

(6) $$\Delta \phi_{\beta \alpha }^{i}=\Delta \rho_{\beta \alpha }^{i}-c\Delta b_{\beta \alpha }-\lambda \Delta N_{\beta \alpha }^{i}+\Delta \epsilon_{\phi ,\beta \alpha }^{i}$$

By subtracting Equation (5) from Equation (6), the clock error term, $\Delta b_{\beta \alpha }$, is cancelled out in the following double difference observation equation:

(7) $$\nabla \Delta \phi_{\beta \alpha }^{ij}=\nabla \Delta \rho_{\beta \alpha }^{ij}-\lambda \nabla \Delta N_{\beta \alpha }^{ij}+\Delta \epsilon_{\phi ,\beta \alpha }^{ij}$$

Note that $\nabla \Delta N_{\beta \alpha }^{ij}$ in Equation (7) converges to a unique integer value only when the rover and the base successfully receive carrier signals from multiple (typically more than 6) satellites [34]. The mode of RTK-GPS in this condition is called to a fixed mode, and RTK-GPS can achieve centimeter accuracy in the fixed mode [35]. However, when the rover and the base cannot reliably receive carrier signals from enough satellites due to signal blockage or weather conditions, the RTK-GPS turns into a floating mode and the accuracy of RTK-GPS is deteriorated down to 10–50 cm. In particular, a serious baseline wandering, where low frequency noises are highly amplified, occurs in displacement estimation with the floating mode.

The measurement quality of RTK-GPS can be further enhanced by adopting higher-grade GPS antennas, which support APC compensation and multipath rejection. It is also preferred to choose GPS receivers and antennas, which support multi-constellation and multi-frequency. Multi-constellation receivers and antennas can access not only GPS satellite signals but also GLONASS, Galileo and BeiDou satellite signals [36]. The use of multi-frequency significantly improves displacement measurement accuracy by utilizing multiple carrier signals (i.e., L1, L2 and L5 signals) with different frequencies (carrier wave length) [37]. However, these GPS receivers and antennas are expensive (around $100,000 USD per unit).

Standard GPS can also measure velocity with 10 cm/s accuracy [38]. The velocity of a GPS receiver is determined by differentiating the present position measurement with and the immediately preceding position measurement. Because of the satellite and GPS receiver clock inaccuracy, and the fact ionospheric and tropospheric delays are cancelled out during the differentiation process, the accuracy of velocity measurement is greatly improved. The estimate of the distance between a satellite and a receiver can be expressed as:

(8) $$\rho \left(t\right)=r\left(t\right)+c\left(b-b_{sat}\right)+I-Tr+\epsilon_{\phi }$$

where r(t) is the true distance between the satellite and the rover. When Equation (8) is differentiated with respect to time, $b_{sat}$, I and Tr terms are cancelled out:

(9) $$\Delta \rho \left(t\right)=\Delta r\left(t\right)+c\Delta b+\Delta \epsilon_{\phi }=\left(v_{sat}\left(t\right)-v\left(t\right)\right)e\left(t\right)+c\Delta b+\Delta \epsilon_{\phi }$$

where $v_{sat}\left(t\right)$ and v(t) are the velocities of the satellite and the rover, respectively, and e(t) is a unit vector whose direction is from the rover to the satellite. Then, v(t) can be obtained from Equation (9). Note that velocity is measured solely from the rover without any input from the base.

The velocity of a GPS receiver also can be calculated by obtaining relative motion of a GPS sensor to a satellite using the frequency of a satellite signal from the satellite [39]. Let $f_{t}$ and $f_{r}$ be the frequencies of transmitted and received satellite signals, respectively, then frequency changes $\Delta f_{d}$ due to Doppler effect is:

(10) $$\Delta f_{d}=f_{r}-f_{t}=-\frac{f_{t}}{c}\left[\left(v_{s}-v_{r}\right)*\frac{r_{s}-r_{r}}{∥r_{s}-r_{r}∥}\right]$$

where $r_{s}$ and $v_{s}$ are the satellite position and velocity vectors, and $r_{r}$ and $v_{r}$ are the receiver position and velocity vectors, respectively. Let drift rate ${\dot{t}}_{r}=\left(f_{r}-f_{t}\right)/f_{t}$, and substituting ${\dot{t}}_{r}$ into Equation (10), pseudorange rate ${\dot{\rho }}_{s}$ can be obtained as:

(11) $${\dot{\rho }}_{s}=\left(v_{s}-v_{r}\right)*a_{s}+c\delta {\dot{t}}_{r}+\epsilon_{{\dot{\rho }}_{s}}$$

where $a_{s}=r_{s}-r_{r}/∥r_{s}-r_{r}∥$, $\delta {\dot{t}}_{r}$ is a receiver clock drift and $\epsilon_{{\dot{\rho }}_{s}}$ is an observation error. Here, $v_{s}$ is a known value since it can be calculated from GNSS ephemeris. Then, $v_{r}$ and $c\delta {\dot{t}}_{r}$ can be estimated via Kalman filter or least squares filter.

## 3. The Proposed Displacement Measurement System

**Figure 1:**
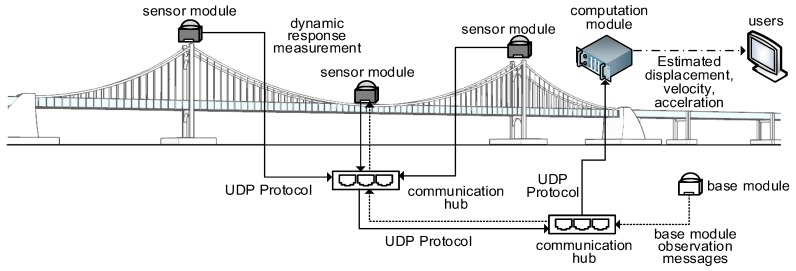
Overview of the proposed measurement system including sensor, base and computational modules.

In Section 3, the proposed displacement measurement system is introduced in details. Figure 1 shows the overall configuration of the proposed displacement measurement system. The measurement system is composed of a base module, a sensor module and a computational module. The base module in Figure 1 is necessary for initial displacement measurement using RTK-GPS technology. The so-called ‘observation messages’ containing measurement timestamp and absolute position information is created at the base module and transmitted to the sensor module using a user datagram protocol (UDP) communication protocol through optical fibers. The sensor modules are installed at multiple measurement points over the target structure where displacement measurements are necessary. The sensor module consists of a GPS receiver, a 3-axis force feedback accelerometer, an accelerometer control board, an analog digital converter (ADC) board, and a micro controller unit (MCU) board. The initial displacement is measured using the GPS receiver in the sensor module and the observation messages from the GPS base module. In addition, the velocity is measured solely from the GPS receiver in the sensor module, and the acceleration is measured by the accelerometer. All the measurements are time synchronized and transmitted to the computation module using the same UDP protocol. The computation module will be often installed at a central monitoring facility. The computation module estimates 3D displacements, velocities and accelerations by employing a series of data processing techniques including a two-stage Kalman filter.

### 3.1. Base Module

The base module generates observation messages including GPS clock time, ϕ and λN in Equation (1) and a signal to noise ratio, and continuously transfers the observation messages to the sensor module using the UDP protocol as illustrated in Figure 1.

**Figure 2:**
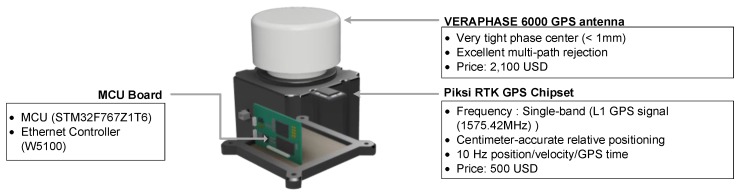
The base module composed of a GPS antenna and a GPS chipset.

The base module consists of a Piksi RTK-GPS chipset (SwiftNav, San Francisco, CA, USA) a VERAPHASE 6000 GPS antenna (Tallysman, Ottwa, Canada), a MCU board for data transmission and an Ethernet controller (Figure 2). The RTK-GPS chipset is chosen because of its cost effectiveness (around $500 USD). On the other hand, the VERAPHASE 6000 is chosen in spite of its high price (around $2100 USD) since this antenna has an excellent antenna center tightness (1 mm) and can alleviate the multipath problem. Note that a high-end antenna is used at the base because the quality of displacement measurements at all sensor modules heavily depends on the quality of the observation messages from the base. The base module is installed on a fixed reference point, and the movement of the base is assumed to be negligible. It is recommended that the GPS base be installed as close as possible to the sensor modules. As a rule of thumb, the displacement accuracy deteriorates about 1–2 mm/km as the distance between the base and the sensor module increases. Often two base modules are employed for improved accuracy and reliability.

### 3.2. Sensor Module

**Figure 3:**
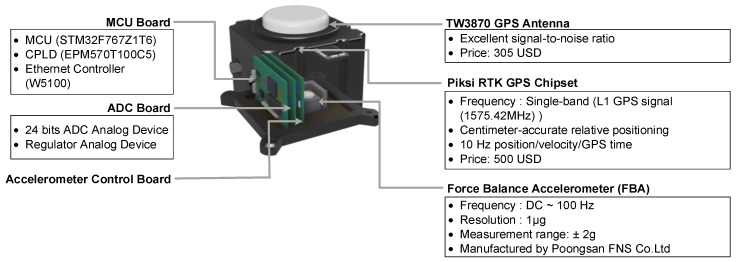
The sensor module composed of a GPS antenna, GPS chipset and a force feedback accelerometer.

The sensor module is composed of a 3-axis force feedback accelerometer, a GPS antenna, a GPS chipset, an ADC board, an acceleration control board and a MCU board with a complex programmable logic device (CPLD) and an Ethernet controller as shown in Figure 3. The MCU board controls all the components in the sensor module, and synchronizes all measurements. Then, all the information, such as GPS time, acceleration, velocity, displacement, number of satellites, and fixed/float flag, generated by the sensor module is transmitted to the computation module. The sensor module requires a +5V DC power supply for operation, and optical fibers and UDP protocol are used for data transmission between the sensor module and the computation module.

In this study, a 3-axis force feedback accelerometer is designed and manufactured by Poongsan FNS Co. (Nonsan, Korea) specifically for infrastructure applications. The accelerometer has 120 dB dynamic range of, ±2 g measurement range, DC to 100 Hz frequency range, and 1000 μg/g^2^ nonlinearity. Acceleration responses are digitized by a 24-bit ADC chip (AD7779ACPZ, Analog Devices, Norwood, MA). Note that the force feedback accelerometer has a strong bias stability compared to MEMS accelerometers and a better performance in a frequency range of DC to 10 Hz.

Like the base module, the SwiftNav Piksi RTK-GPS receiver is used in the sensor module. On the other hand, a TW3870 GPS antenna (Tallysman, Ottwa, Canada) is adopted in the sensor module instead of the VERAPHASE 6000 antenna because of its low cost (around 300 USD). Using the RTK-GPS technology, the GPS receiver in the sensor module computes the RTK displacement using the observation messages from the base module.

Note that the velocity measurement is solely from the GPS receiver in the sensor module and independent of the GPS base module. The GPS receiver sends out displacement, velocity, GPS time, number of satellites, and Fixed/Float flag information to the MCU at every 100 ms (10 Hz). Then, the MCU chip generates a data packet and puts the information obtained from the GPS receiver into the data packet.

**Figure 4:**
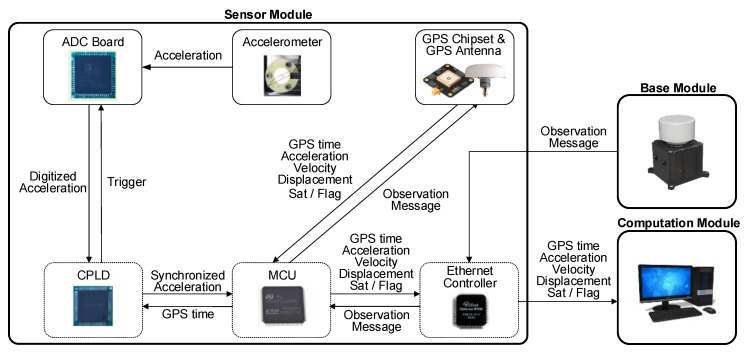
Block diagram of sensor module operation.

As illustrated in Figure 4, the accelerometer response is digitized by a 24-bit ADC board (AD7779ACPZ) at 100 Hz. After ADC, the digitized acceleration and its timestamp are transmitted to the MCU board and merged into the data packet. Then, the data packet is transferred to the computation module through an Ethernet controller in the sensor module. Note that the ADC is triggered by the CPLD at every 10 ms (100 Hz) with a maximum delay of 5.6 ns, and the CPLD is synchronized with GPS time at every 100 ms (10 Hz) with a maximum error of 60 ns. Therefore, the overall time synchronization error between acceleration and displacement measurements is expected to be less than 0.1 ms.

**Figure 5:**
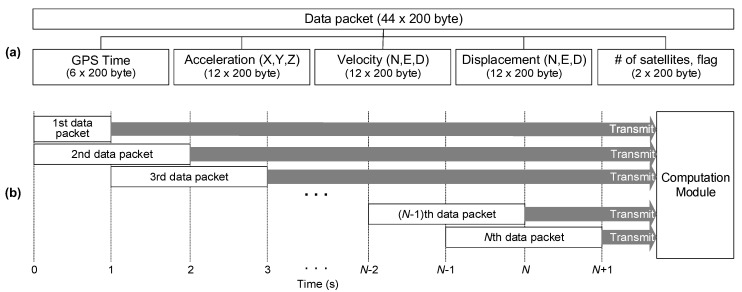
The data packet produced in the sensor module: (**a**) data packet structure; and (**b**) data packet transmission process.

A single data packet includes the information generated by the sensor module for 2 s. To be more specific, a single data packet consists of 200 samples of timestamp and acceleration response and 20 samples of velocity, displacement, number of satellites, and fixed/float flag, resulting in a data packet with 8800 bytes as shown in Figure 5a. The 2 s data packet is transmitted to the computation module at every 1 second using UDP. Note that UDP is selected in this study instead of transmission control protocol (TCP), because UDP allows real-time, unicast, multicast and broadcast data transmissions while TCP allows only unicast transmission. Typically, UDP can transmits data at a maximum speed of a specific network, and has a low data transmission overhead. However, this real-time data transmission is achieved at the expense of omitting error checking, and makes UDP vulnerable to data loss. The typical data transmission reliability of UDP is 99% or higher. To avoid data loss, the data in the second half of a single data packet is copied into the first half of the next data packet and transmitted again. In this way, all the data is transmitted twice as shown in Figure 5b. For example, even when the transmission of the second data packet fails, the data for 0 to 2 s still can be retrieved from the first and third data packets.

### 3.3. Computation Module

**Figure 6:**
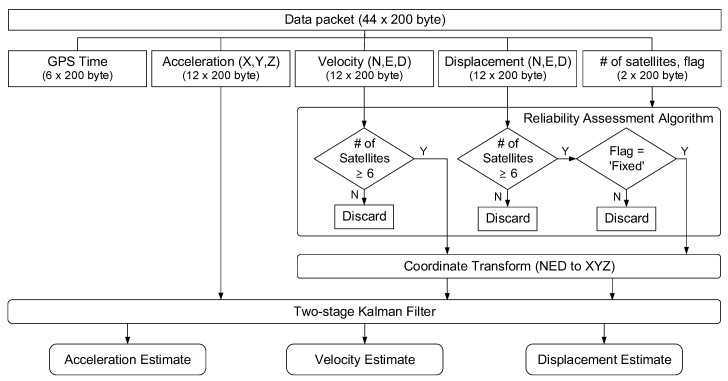
Data processing flowchart for dynamic response estimation at computation module.

Once the data packets are received from the sensor module, the reliability of the displacement and velocity data is evaluated by a reliability assessment algorithm, and the measured displacement and velocity are combined with the measured acceleration using a two-stage Kalman filter for high-accuracy displacement estimation, as shown in Figure 6. Kim et al. [28] demonstrated through a series of numerical simulation that the two-stage Kalman filter can achieve a higher estimation accuracy without smoothing and better robustness under various noise levels than Smyth and Wu [25] and Kim et al. [26]. Note that all the response estimation for each step should be completed within 10 ms because the data is sampled at 100 Hz. Using a personal computer with 2.5 GHz CPU, 8 GB memory, and 100 Mbps Ethernet port, the response estimation for each time step took less than 1 ms. Therefore, it may be possible to simultaneously process data from ten sensor modules. If data from more than ten sensor modules needed to be processed simultaneously, a multi-threading algorithm could be employed to reduce the total processing time for all the sensor modules [40].

The first step in the computational module is the reliability assessment of RTK-GPS data. As mentioned in Section 2, the reliability of GPS data heavily depends on (1) from how many satellites each sensor module can receive carrier signals, and (2) whether the estimated ambiguity integer is converged. In general, as each sensor module can receive carrier signals from more satellites, the reliability of the measurement is improved. For the GPS-RTK chipset adopted in the proposed system, the authors’ experience indicates that reliable velocity measurement is possible only when the number of satellites is greater than or equal to 6.

When the number of satellites becomes less than 6, the quality of velocity measurement deteriorates drastically, and the measurement is intermittently interrupted. In this study, the displacement and the velocity measured from the sensor module is used for the subsequent displacement estimation in the Kalman filter if the sensor module can receive carrier signals from six or more satellites. Otherwise, the displacement and the velocity measurement are disregarded for displacement estimation. In addition, the displacement measured by RTK-GPS can be proceeded with the Kalman filter only when the flag indicator turns into a fixed mode. Otherwise, the displacement measurement is disregarded as well. Then, the coordinates of displacement and velocity are aligned with the coordinates of acceleration, and the displacement and velocity measurements along with the acceleration data are proceeded with the Kalman filter.

### 3.4. Displacement Estimation Using Two-Stage Kalman Filter

In this study, a two-stage Kalman filter [41] is adopted to estimate displacement, velocity and acceleration with high accuracy and high sampling rate. In particular, the Kalman filter based data fusion technique proposed by Kim et al. [27] is further advanced to include velocity measurement.

Defining a state vector as $x\left(k\right)={\left[\begin{array}{ccc} x\left(k\right) & \dot{x}\left(k\right) & \ddot{x}\left(k\right) \end{array}\right]}^{T}$, where x(k) is true displacement, $\dot{x}\left(k\right)$ is true velocity, and $\ddot{x}\left(k\right)$ is true acceleration, a transition equation can be expressed as follows:

(12) x(k)=Ax(k−1)+Bw(k−1)

(13) $$A=\left[\begin{array}{ccc} 1 & ∆t & \frac{1}{2}∆t^{2}\\ 0 & 1 & ∆t\\ 0 & 0 & 1 \end{array}\right],\;B=\left[\begin{array}{c} \frac{1}{2}∆t^{2}\\ ∆t\\ 1 \end{array}\right]$$

where ∆t is a sampling interval, and w(k) is a process white noise with covariance, q(k).

Also, an observation equation can be expressed as follows:

(14) y(k)=Hx(k)+Cb(k)+v(k)

where $b\left(k\right)={\left\{\begin{array}{ccc} b_{x} & b_{\dot{x}} & b_{\ddot{x}} \end{array}\right\}}^{T}$ is a bias vector, and $b_{x}$, $b_{\dot{x}}$ and $b_{\ddot{x}}$ are biases contained in the displacement, velocity and acceleration measurements, respectively. $v\left(k\right)={\left\{\begin{array}{ccc} v_{x} & v_{\dot{x}} & v_{\ddot{x}} \end{array}\right\}}^{T}$ is a measurement white noise vector composed of displacement, velocity and acceleration measurement noises, and y(k) is a measurement vector. Note that the observation equation in Equation (14) has different forms at each time step, because acceleration, velocity and displacement measurements have different sampling rates, and available measurements change at each time step. The different forms of the observation equation are summarized in Table 1.

In stage 1, the response of a structure is estimated by ignoring the bias term in the acceleration measurement (i.e., the observation equation in Equation (14) changes to y(k)=Hx(k)+v(k)) and applying a conventional Kalman filter [42]:

(15) $${\tilde{x}}^{-}\left(k\right)=A{\tilde{x}}^{+}\left(k-1\right)$$

(16) $$P_{x}^{-}\left(k\right)=AP_{x}^{+}\left(k-1\right)A^{T}+q\left(k\right)BB^{T}$$

(17) $$K_{x}\left(k\right)=P_{x}^{-}\left(k\right)H^{T}{\left[HP_{x}^{-}\left(k\right)H^{T}+R\left(k\right)\right]}^{-1}$$

(18) $${\tilde{x}}^{+}\left(k\right)={\tilde{x}}^{-}\left(k\right)+K_{x}\left(k\right)\left[y\left(k\right)-H{\tilde{x}}^{-}\left(k\right)\right]$$

(19) $$P_{x}^{+}\left(k\right)=P_{x}^{-}\left(k\right)-K_{x}\left(k\right)HP_{x}^{-}\left(k\right)$$

where A, B, H, and y(k) are the state space matrixes defined in Equations (12) and (14), q(k) is a covariance of w(k), ${\tilde{x}}^{-}\left(k\right)$ and ${\tilde{x}}^{+}\left(k\right)$ are prior and posterior state vector estimates, $P_{x}^{-}\left(k\right)$ and $P_{x}^{+}\left(k\right)$ are prior and posterior error covariance matrices, and $K_{x}\left(k\right)$ is Kalman gain matrix in stage 1, respectively. Note that error covariance matrices are constructed from the variance of the measurements under assumption that each noise process is uncorrelated with others [27]. The proposed system initially measures acceleration, velocity and displacement of a structure in a static condition (zero response condition) before installation on a structure, and then calculates the variances of the measurements.

In Stage 2, the bias omitted in Stage 1 is estimated, and the estimates of the responses are updated considering the bias. Stage 2 starts from an assumption that the responses estimated in Stage 1 are related to the responses in Stage 2 as follows:

(20) $${\hat{x}}^{-}\left(k\right)={\tilde{x}}^{-}\left(k\right)+U\left(k\right)b\left(k\right)$$

(21) $${\hat{x}}^{+}\left(k\right)={\tilde{x}}^{+}\left(k\right)+V\left(k\right)b\left(k\right)$$

where ${\tilde{x}}^{-}\left(k\right)$ and ${\tilde{x}}^{+}\left(k\right)$ are prior and posterior state vector estimates in stage 1, ${\hat{x}}^{-}\left(k\right)$ and ${\hat{x}}^{+}\left(k\right)$ are prior and posterior state vector estimates in stage 2, and U(k) and V(k) are sensitivity matrices, respectively.

Assuming the bias is piecewise constants, it can be expressed as:

(22) b(k)=b(k−1)

**Figure 7:**
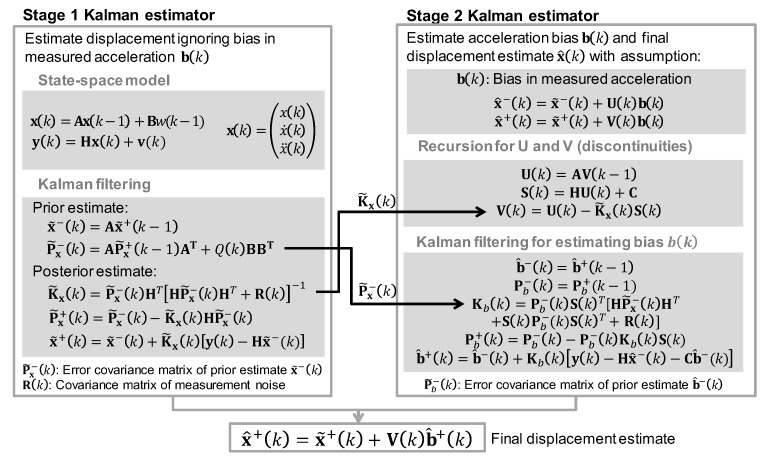
Overview of two-stage Kalman filter algorithm.

Defining the residual vector $r\left(k\right)=y\left(k\right)-{\tilde{x}}^{-}\left(k\right)$ and applying Equation (18), the observation vector in the Stage 2 Kalman filter is expressed as:

(23) r(k)=S(k)b(k)+z(n)

where S(k) is a sensitivity matrix and z(n) is a zero-mean Gaussian random process expressed as $z\left(n\right)=y\left(k\right)-H{\hat{x}}^{+}\left(k\right)$. Equations (22) and (23) establish the state-space model in Stage 2. To summarize, the estimated state vector from Stage 1 is corrected by bias vector from Stage 2, and the improved dynamic responses can be estimated from the updated state vector in Equation (21). The two-stage Kalman filter algorithm is summarized in Figure 7.

## 4. Lab-Scale Experiments

**Figure 8:**
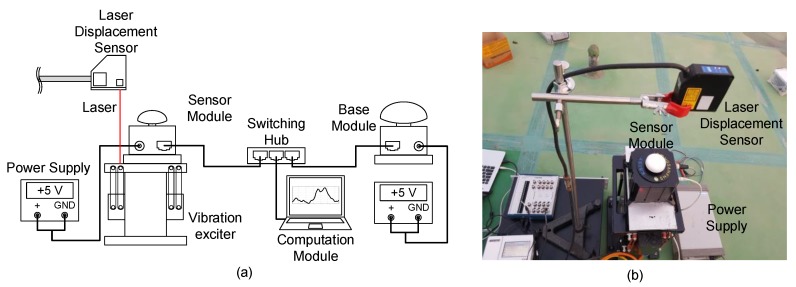
Overview of the lab-scale experiment: (**a**) experiment configuration and; (**b**) sensor module installation.

A series of lab-scale experiments were performed to validate the performance of the proposed system. The overall setup for the vibration tests is shown in Figure 8. A sensor module is attached on an ElectroSeis vibration exciter (APS Dynamics, San Juan Capistrano, CA, USA) such that the sensor module is subjected to vertical vibration. The sensor module is powered by a +5 V DC power supply, and connected to a base module, and a computation module in a personal computer through LAN cables and a switching hub. The base module is installed 5 m away from the sensor module, powered by the same power supply. The reference displacement is measured using a CD5-W500 laser displacement sensor (Optex FA, Kyoto, Japan), which has 10 *μ*m resolution and ±0.08% linearity.

The sensor module was subjected to three types of vibrations. Case 1: 0.3 Hz sinusoidal vibration, Case 2: a random vibration with a frequency band of DC to 10 Hz, and Case 3: no external vibration. In Cases 1 and 2, only the vertical responses are compared because the exciter was mainly vibrated in the vertical direction. The sinusoidal vibration in Case 1 was designed to simulate a typical response of a long-span bridge, and the random vibration represented a structural response under seismic ground motion. Each vibration was applied to the sensor module for 5 min, and the maximum displacement of the sensor module was set within the maximum stroke of the vibration exciter, 10 cm. In addition, to estimate a noise level, the sensor response was measured without any external input in Case 3.

The sensor module measured acceleration, velocity and displacement, and transmitted the measurements to the computation module through a switching hub. The number of satellites continuously observed during the experiment was 9–10. The computation module improved the accuracy of the acceleration, velocity and displacement by fusing the measurements received from the sensor module using the two-stage Kalman filter.

### 4.1. Case 1–0.3 Hz Sinusoidal Vibration

**Figure 9:**
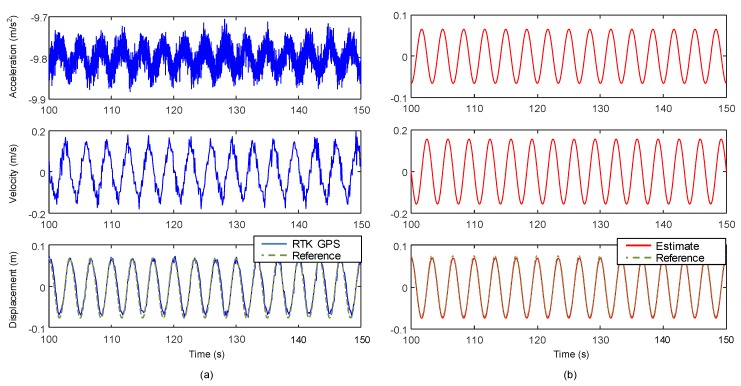
Comparison of measurements before and after applying two-stage Kalman filter (for 0.3Hz sinusoidal vibration input in Case 1): (**a**) initial acceleration from accelerometer, velocity from GPS, displacement from RTK-GPS before applying Kalman filter; (**b**) corresponding estimated responses from the proposed measurement system after applying Kalman filter.

Test results for Case 1 are presented in Figure 9. Figure 9a shows acceleration obtained from the accelerometer in the sensor module, velocity from the GPS chipset, and displacement measured by RTK-GPS before applying a two-stage Kalman filter. Figure 9b shows the corresponding responses obtained from the proposed measurement system after applying the two-stage Kalman filter. The RMSE of the displacement was reduced from 10.45 to 1.13 mm (by 89.19%) using the two-stage Kalman filter.

**Figure 10:**
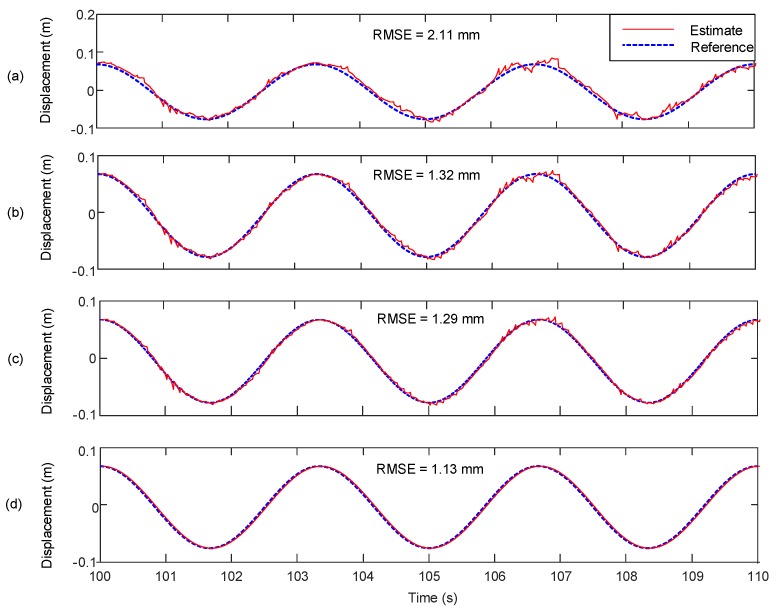
Comparison of the proposed measurement system with other data fusion techniques (for 0.3 Hz sinusoidal vibration input in Case 1: (**a**) Smyth and Wu [24] with RTS smoothing; (**b**) Kim et al. [25] with forward-backward smoothing; (**c**) Kim et al. [27] and; (**d**) the proposed system.

The displacement estimation performance of the proposed system is compared with those of Smyth and Wu [24] and Kim et al. [25] in Figure 10. Because the acceleration bias is not considered in Smyth and Wu [24], the error produced by double integration of acceleration is accumulated over time as shown in Figure 10a. The acceleration bias is considered, and the associated error is removed in Kim et al. [25] shown in Figure 10b. However, there is a remaining high frequency error caused by sudden corrections of acceleration measurement bias in the posterior step of Kalman filtering. The proposed system explicitly considers the acceleration bias and removes the low frequency drift and the high frequency error using additional velocity measurement as shown in Figure 10d. The proposed system improves the displacement estimation accuracy by 46.45% (from 2.1 to 1.13 mm) and 14.39% (from 1.32 to 1.13 mm) compared to Smyth and Wu [24] and Kim et al. [25], respectively. In addition, real-time estimation is difficult for Smyth and Wu [24] and Kim et al. [25], because additional smoothing demands more computational time and causes time delays [26]. In the Figure 10, RTS and forward-backward smoothing algorithms are applied to Smyth and Wu [24] and Kim et al. [25], respectively, since these algorithms were adopted in the references. However, the proposed system does not need any smoothing, making it attractive for real-time displacement. In the lab-scale test, response estimation at each time step took less than 1 ms using the proposed system.

In Figure 10c,d the effect of velocity measurement on displacement estimation using the proposed system is examined for 0.3 Hz sinusoidal vibration input in Case 1. Kim et al. [27], which uses two-stage Kalman filter without using the velocity measurement, cannot remove the low frequency drift and high frequency noise properly as shown in Figure 10c. When the velocity measurement was used in addition to the acceleration and displacement measurements, the low frequency drift and high frequency noise was alleviated and the RMSE was reduced from 1.29 to 1.13 mm (by 12.40%).

### 4.2. Case 2—A Random Vibration with a Frequency Band of DC to 10 Hz

**Figure 11:**
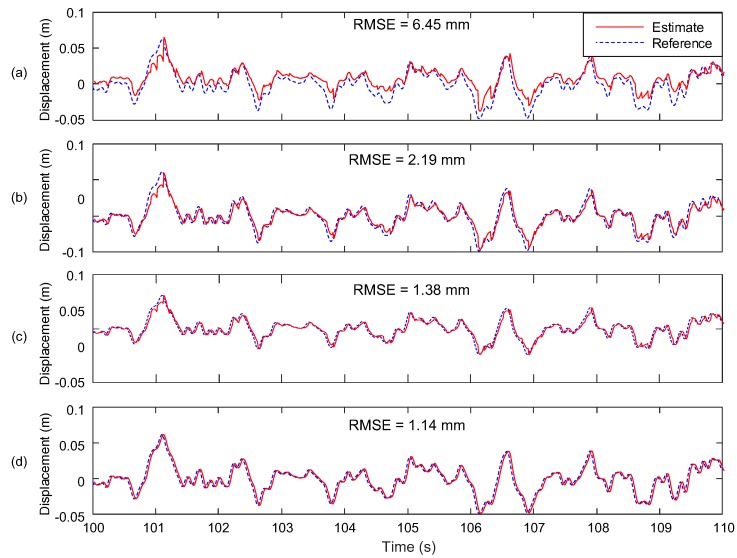
Comparison of RMSE of the proposed system with other Kalman filter based displacement estimation techniques (for a random vibration in Case 2): (**a**) Smyth and Wu [24]; (**b**) Kim et al. [25]; (**c**) Kim et al. [27] and; (**d**) the proposed method.

Figure 11 compares the displacement estimation performance of the proposed system with Smyth and Wu [24], Kim et al. [25], and Kim et al. [27] for Case 2. The proposed system reduces the RMSE by 82.33% (from 6.45 to 1.14 mm) with respect to Smyth and Wu [24], and 47.95% (from 2.19 to 1.14 mm) with respect to Kim et al. [25], respectively. In addition, consideration of velocity measurement into the two-stage Kalman filter reduces the RMSE by 17.39% (from 1.38 to 1.14 mm).

### 4.3. Case 3—No External Vibration

In Case 3, no external vibration is applied to the sensor module to investigate the effect of the proposed measurement system on noise reduction. Because no external vibration is applied, the responses in X, Y and Z directions should be close to zero and the RMSE is calculated as a root mean square of the measurements. The noise reduction effect is examined using the following definition:

(24) $$\mathrm{Noise}\;\mathrm{reduction}\left(\%\right)=\frac{\mathrm{RMSE}\left(\mathrm{ACC}\;\mathrm{or}\;\mathrm{GPS}\;\mathrm{sensor}\right)-\mathrm{RMSE}\left(\mathrm{Proposed}\;\mathrm{system}\right)}{\mathrm{RMSE}\left(\mathrm{ACC}\;\mathrm{or}\;\mathrm{GPS}\;\mathrm{sensor}\right)}\times 100$$

The effect of the proposed measurement system on noise reduction is summarized in Table 2. The proposed system reduced the noise level compared to the raw measurements from the accelerometer or the GPS chipset. On average 71.03% noise reduction in acceleration, 62.40% in velocity and 81.95% in displacement are achieved considering all three axes.

## 5. Field Tests

The performance of the proposed measurement system has been examined through field tests at the Yeongjong Grand Bridge and Yi Sun-sin Bridge in South Korea, and the Xihoumen Bridge in China. First, test results from the Yeongjong Grand Bridge are presented. The Yeongjong Grand Bridge is a self-anchored suspension bridge which has a 300 m-long main span and two 120 m-long side spans. The bridge has double-layered steel truss decks. The upper deck is for regular vehicle traffic and the bottom deck for high-speed trains and vehicles. Typically, 6 to 7 cm deflection is observed at its middle span when the 692-ton train crosses the bridge.

**Figure 12:**
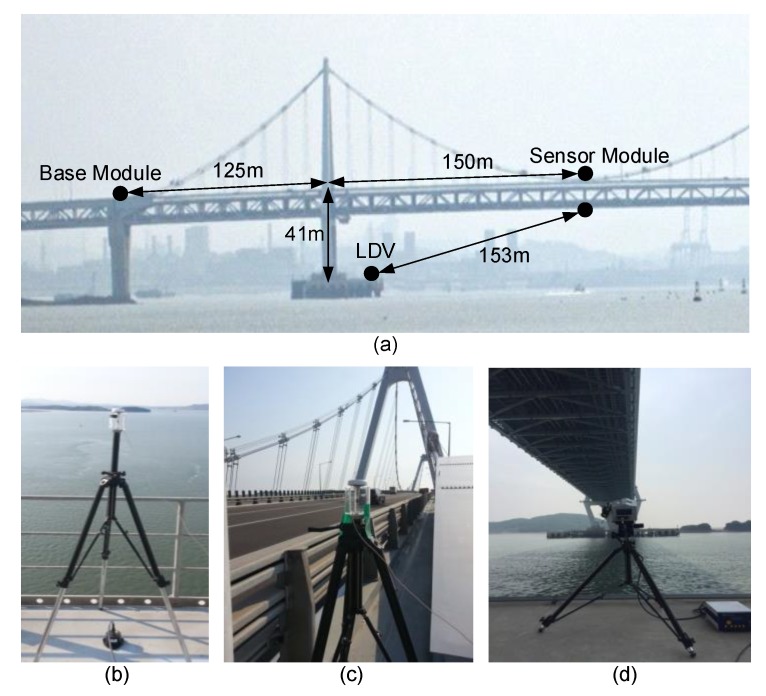
System configuration for the displacement measurement at Yeongjong Grand Bridge: (**a**) locations of sensors; (**b**) sensor module; (**c**) base module and; (**d**) LDV for reference displacement measurement.

In the experiment, a sensor module and a base module were installed as described in Figure 12. The sensor module was installed in the middle of the main span, and the base module was placed on the top of a pier, as shown in Figure 12a. In a preliminary experiment using a 3713E112G 3-axis MEMS DC accelerometer (PCB Piezotronics, Depew, NY, USA) and an LF-24 low-frequency geophone (ION, Houston, TX, USA), no significant structural response was observed at the base module installation point. A reference displacement was measured by a RSV-150 laser Doppler vibrometer (Polytec, Waldbronn, Germany) placed on one of the pylon foundations, as shown in Figure 12d.

**Figure 13:**
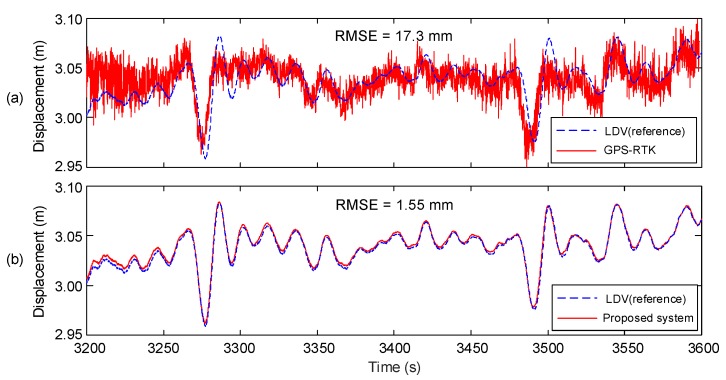
Comparison of (**a**) the displacement measured by RTK-GPS and; (**b**) the displacement estimated by the proposed measurement system.

The displacement measured by a RTK-GPS sensor and the displacement estimated by the proposed system are compared with the LDV reference displacement in Figure 13. Note that 7–9 satellite signals were received during the experiments. The accuracy of displacement estimation was improved from 17.33 to 1.55 mm (91.06%) using the proposed system. Furthermore, Figure 13 shows that the proposed measurement system can effectively remove high frequency noise components and properly trace a low frequency vibration trend.

**Figure 14:**
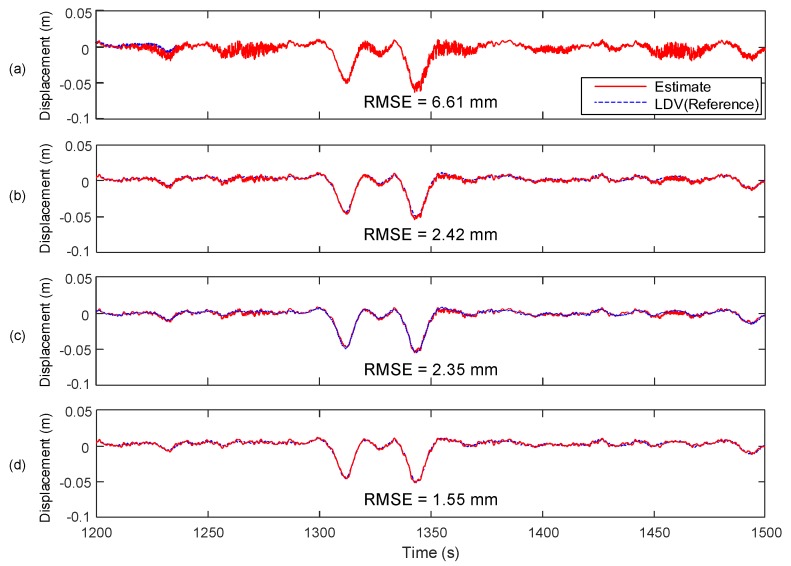
Comparison of the proposed method with other data fusion techniques: (**a**) Smyth and Wu [24]; (**b**) Kim et al. [25]; (**c**) Kim et al. [27] and; (**d**) the proposed system.

The displacement estimation performance of the proposed system is compared with those of Smyth and Wu [24], Kim et al. [25] and Kim et al. [27] in Figure 14. The proposed system reduces the RMSE of displacement estimation by 76.55% (from 6.61 to 1.55 mm) with respect to Smyth and Wu [24] and 35.95% (from 2.42 to 1.55 mm) with respect to Kim et al. [25], respectively. Comparison of the proposed system with to Kim et al. [27] reveals that the inclusion of velocity measure into the two-stage Kalman filter can further reduce RMSE of displacement from 2.35 to 1.55 mm (by 34.04%).

Additional field tests with a similar experiment setup were conducted at the Yi Sun-sin Bridge in South Korea, which is a suspension bridge having 1545 m-long main span, and the Xihoumen Suspension Bridge in China, with a 1650 m-long main span. The test results for all three bridges including the Yeongjong Grand Bridge are summarized in Table 3. The proposed system improves displacement estimation accuracy compared to the other data fusion techniques. On average 29.91% RMSE reduction compared to Kim et al. [27], 42.66% RMSE reduction compared to Kim et al. [25], and 82.63% RMSE reduction compared to Smyth and Wu [24] are achieved.

## 6. Conclusions

In this paper, a high-fidelity displacement measurement system is proposed for continuous and real-time monitoring of large-scale civil infrastructure. The authors have concluded that the performance and cost of the proposed displacement measurement systems is quite appealing compared to conventional RTK-GPS sensors. The proposed system estimates 3-axis displacement, velocity and acceleration with a high accuracy of 2 mm and a high sampling rate of 100 Hz by fusing the acceleration measured from a force feedback accelerometer, and the velocity and displacement measured from a GPS chipset. The accelerometer and the GPS chipset are integrated into a single sensor module, and the sensor module is installed on a target structure like conventional RTK-GPS sensors. Besides the sensor module, the proposed displacement measurement system is composed of a base module and a computational module. The performance of the proposed system was validated through lab-scale and field bridge tests. Average RMSE of estimated displacements was 1.14 mm and 1.64 mm for the lab-scale tests and the field tests, respectively. In addition, the noises in acceleration, velocity and displacement were reduced by 71.03%, 62.40% and 81.95%, respectively. A follow-up study is underway to improve the displacement measurement accuracy by adopting a low-cost multi-frequency (L1, L2 and L5) and multi-constellation (GPS, GLONASS, Galileo and Beidu) RTK-GPS receiver. Furthermore, the proposed system is being updated so that displacement can be measured even during seismic events when RTK-GPS signal is compromised due to the movement of the base sensor.

## Figures and Tables

**Table 1:** Variation of observation equations depending on the availability of reliable measurements.

Available Measurements				Observation Equation of State-Space Model			
Reliability Assessment	Displacement (from RTK GPS)	Velocity (from GPS Sensor)	Acceleration (from Accelerometer)	y(k)	H	C	v(k)
#sat ≥ 6 Flag = ‘Fixed’	◯	◯	◯	$\left[\begin{array}{c} x_{m}\left(k\right)\\ {\dot{x}}_{m}\left(k\right)\\ {\ddot{x}}_{m}\left(k\right) \end{array}\right]$	$\left[\begin{array}{cc} 1 & \begin{array}{cc} 0 & 0 \end{array}\\ \begin{array}{c} 0\\ 0 \end{array} & \begin{array}{cc} \begin{array}{c} 1\\ 0 \end{array} & \begin{array}{c} 0\\ 1 \end{array} \end{array} \end{array}\right]$	$\left[\begin{array}{c} 0\\ 0\\ 1 \end{array}\right]$	$\left[\begin{array}{c} v_{x}\left(k\right)\\ v_{\dot{x}}\left(k\right)\\ v_{\ddot{x}}\left(k\right) \end{array}\right]$
#sat ≥ 6 Flag = ‘Float’	☓	◯	◯	$\left[\begin{array}{c} {\dot{x}}_{m}\left(k\right)\\ {\ddot{x}}_{m}\left(k\right) \end{array}\right]$	$\left[\begin{array}{cc} \begin{array}{cc} 0 & 1 \end{array} & 0\\ \begin{array}{cc} 0 & 0 \end{array} & 1 \end{array}\right]$	$\left[\begin{array}{c} 0\\ 1 \end{array}\right]$	$\left[\begin{array}{c} v_{\dot{x}}\left(k\right)\\ v_{\ddot{x}}\left(k\right) \end{array}\right]$
#sat < 6	☓	☓	◯	$\left[{\ddot{x}}_{m}\left(k\right)\right]$	$\left[\begin{array}{ccc} 0 & 0 & 1 \end{array}\right]$	[1]	$\left[v_{\ddot{x}}\left(k\right)\right]$

**Table 2:** The effect of the proposed measurement system on noise reduction in X, Y and Z axes.

Dynamic Response	Axis	RMSE		
		ACC or GPS Sensor	Proposed System	Noise Reduction (%)
**Acc. (m/s^2^)**	X	0.170	0.044	74.12
	Y	0.159	0.041	74.21
	Z	0.184	0.065	64.68
	Avg.	0.171	0.050	71.03
**Vel. (mm/s)**	X	21.235	7.101	66.56
	Y	23.481	8.895	62.12
	Z	32.734	13.574	58.53
	Avg.	25.817	9.857	62.40
**Disp. (mm)**	X	5.776	1.010	82.51
	Y	6.539	1.484	77.31
	Z	11.527	1.610	86.03
	Avg.	7.947	1.368	81.95

**Table 3:** Comparison of displacement estimation performances through field bridge tests.

Test Sites	RMSE (mm)			
	The Proposed System	Kim et al. [27]	Kim et al. [25]	Smyth and Wu [24]
**Yeongjong Grand Bridge**	1.55	2.35	2.42	6.61
**Yi Sun-sin Bridge**	1.23	1.87	3.20	13.38
**Xihoumen Bridge**	2.15	2.81	2.97	8.33
**Avg.**	1.64	2.34	2.86	9.44
